# Many Globally Isolated AD Hybrid Strains of Cryptococcus neoformans Originated in Africa

**DOI:** 10.1371/journal.ppat.0030114

**Published:** 2007-08-17

**Authors:** Anastasia P Litvintseva, Xiaorong Lin, Irka Templeton, Joseph Heitman, Thomas G Mitchell

**Affiliations:** Department of Molecular Genetics and Microbiology, Duke University Medical Center, Durham, North Carolina, United States of America; Johns Hopkins University School of Medicine, United States of America

## Abstract

Interspecific and intervarietal hybridization may contribute to the biological diversity of fungal populations. Cryptococcus neoformans is a pathogenic yeast and the most common fungal cause of meningitis in patients with AIDS. Most patients are infected with either of the two varieties of *C. neoformans,* designated as serotype A *(C. neoformans* var. *grubii)* or serotype D *(C. neoformans* var. *neoformans)*. In addition, serotype AD strains, which are hybrids of these two varieties, are commonly isolated from clinical and environmental samples. While most isolates of serotype A and serotype D are haploid, AD strains are diploid or aneuploid, and contain two sets of chromosomes and two mating type alleles, *MAT**a*** and *MATα,* one from each of the serotypes. The global population of serotype A is dominated by isolates with the *MATα* mating type (Aα); however, about half of the globally analyzed AD strains possess the extremely rare serotype A *MAT**a*** allele (A**a**). We previously described an unusual population of serotype A in Botswana, in which 25% of the strains contain the rare *MAT**a*** allele. Here we utilized two methods, phylogenetic analysis of three genes and genotyping by scoring amplified fragment length polymorphisms, and discovered that AD hybrid strains possessing the rare serotype A *MAT**a*** allele (genotype A**a**Dα) cluster with isolates of serotype A from Botswana, whereas AD hybrids that possess the *MAT*α serotype A allele (AαD**a** and AαDα) cluster with cosmopolitan isolates of serotype A. We also determined that AD hybrid strains are more resistant to UV irradiation than haploid serotype A strains from Botswana. These findings support two hypotheses: (i) A**a**Dα strains originated in sub-Saharan Africa from a cross between strains of serotypes A and D; and (ii) this fusion produced hybrid strains with increased fitness, enabling the Botswanan serotype A *MAT**a*** genome, which is otherwise geographically restricted, to survive, emigrate, and propagate throughout the world.

## Author Summary

Hybridization between individuals of different species or varieties is common among fungi. However, the impact of hybridization on the evolution of pathogenic fungi is unresolved. Several hybrids of phytopathogenic fungi exhibit expanded host ranges. To our knowledge, this report is the first description of increased hybrid fitness (hybrid vigor) in a human pathogen, *Cryptococcus neoformans,* the most prevalent cause of fungal meningitis. We demonstrate that diploid hybrid strains are common among both environmental and clinical isolates of two varieties, represented by serotypes A and D. We determined that many globally isolated AD hybrid strains originated in sub-Saharan Africa and have increased resistance to ultraviolet radiation. We hypothesize that hybrid strains have increased fitness, which enabled them to emigrate from Africa and spread globally.

## Introduction

The impact of hybridization between fungal species and varieties on their evolution is unresolved. Hybridization may be considered an evolutionary disadvantage because some interspecies hybrids have reduced fitness [1,2]. Alternatively, natural hybridization may be beneficial because it can generate new evolutionary lineages that are able to occupy novel ecological niches [2–5]. In recent years, several examples of epidemiologically successful interspecific hybrids that were able to colonize new environments and infect new hosts have been described among fungal plant pathogens [5–7] and oomycetes [4]. These hybrids illustrate the effect of natural hybridization on the production of biological diversity in fungal populations.

C. neoformans is an opportunistic human pathogen that is acquired exogenously and readily isolated from the environment worldwide [8]. Based on serological differences in capsular epitopes and molecular phylogenetic evidence, two varieties are recognized: C. neoformans var. *grubii,* which encompasses isolates of serotype A, and *C. neoformans* var. *neoformans,* which includes isolates of serotype D [8–10]. These varieties represent monophyletic lineages that diverged approximately 18 million years ago [11,12], and according to the phylogenetic species concept, they may reflect cryptic species [13]. More than 90% of clinical isolates from patients with cryptococcosis are strains of serotype A. Strains of serotype D are also found globally, but they are more prevalent in Europe [14]. The clinical manifestations of human infections with serotype A or D appear to be similar, but experimental infections suggest that strains of serotype A are more virulent than strains of serotype D [8,15].

AD strains are hybrids of the two varieties. Whereas most isolates of serotypes A and D are haploid, AD strains are diploid or aneuploid, contain two sets of chromosomes, and possess two mating type alleles, one from each of the two serotype A and D haploid genomes [16–18]. Recent reports suggest that hybrid AD strains may be more common in clinical samples then previously appreciated. For example, a prospective survey of cryptococcosis in Europe from 1997 to 2001 found that up to 30% of all isolates of C. neoformans from patients in Europe were AD hybrids [14]. Strains of both serotype A and serotype D, as well as AD hybrids, are found in the environment, where they are primarily associated with avian feces. Our recent analysis of environmental and clinical populations of C. neoformans in North America revealed that approximately 7.5% of strains isolated from the environment are AD hybrids [19].

C. neoformans has a bipolar mating system with two alternative mating type alleles, *MAT**a*** or *MATα*. When sexual reproduction is induced in the laboratory, haploid *MAT**a*** and *MATα* strains of serotype A or serotype D are capable of plasmogamy, karyogamy, and meiosis, during which they produce dikaryotic hyphae, basidia, and chains of haploid basidiospores. Although most naturally occurring AD hybrid strains are incapable of mating with strains of the opposite mating type, some AD hybrid strains are self-fertile. That is, when stimulated by growth on mating medium in the absence of a mating partner, they produce hyphae, basidia, and basidiospores [16,20]. Most of the basidiospores produced by these self-fertile AD hybrid strains fail to germinate, which suggests that meiosis is impaired in these hybrids. However, approximately 5% of these spores germinate to produce viable diploid, aneuploid, and rarely, haploid cells [16,20,21]. In addition, postzygotic reproductive isolation of serotype A and serotype D is supported by phylogenetic analyses of multiple gene genealogies, which is consistent with the monophyletic origins of both serotypes [11,12,22], and by a comparative genomics analysis of representative strains of serotype A and serotype D, which confirmed that recombination is rare between these serotypes [23].

Clinical and environmental populations of both serotype A and serotype D are dominated by isolates with the *MATα* mating type. Isolates of serotype D with the rare *MAT**a*** mating type have been known for some time [8], and recently, a few strains of serotype A with the *MAT**a*** allele have been isolated from Tanzania [24,25], Italy [26], Hungary [27], and Botswana [28]. However, with the exception of the unique subpopulation of serotype A in Botswana (designated VNB strains), in which 25% of the isolates possessed the *MAT**a*** allele, the prevalence of the *MAT**a*** allele among global isolates of serotype A is less than 0.1% [22].

Although the *MAT**a*** allele is extremely rare among global isolates of serotype A, the *MAT**a*** serotype A allele is common among AD hybrids. Several reports indicate that from 20% to 80% of AD hybrids contain the A**a**Dα genotype [16–19,29]. At least two hypotheses have been proposed to explain the rarity of serotype A *MAT**a*** allele among strains of serotype A but its high prevalence in serotype AD hybrids [16,19,24]: (i) nearly all serotype A isolates with the *MAT**a*** allele are extinct, and AD hybrids represent historic evidence that strains with the *MAT**a*** allele were once more abundant; and (ii) isolates of serotype A with the *MAT**a*** allele are extant, but they are geographically isolated, exist in a different natural reservoir, and/or are nonpathogenic; consequently, they are rarely recovered from environmental samples or clinical specimens.

Our recent discovery of the VNB subpopulation of serotype A in Botswana, which includes a significant number of isolates with the *MAT**a*** allele [22,28], invokes an alternative hypothesis about the origin of A**a**Dα hybrids. These hybrids may have originated from sexual encounters between one or more strains of serotype D and one or more VNB strains of serotype A with the *MAT**a*** allele. The progeny of this union may have enjoyed increased fitness compared to the parental strains, enabling them to expand clonally and disseminate beyond Africa. To test this hypothesis, we investigated whether serotype A *MAT**a*** alleles in non-Botswanan AD hybrids could have originated from the VNB subpopulation of serotype A, and whether hybrid strains are fitter than strains of serotype A or serotype D.

## Results

### Half of AD Hybrid Strains Isolated from Three Continents Possess the Rare Serotype A *MAT**a*** Allele

**Figure 1:**
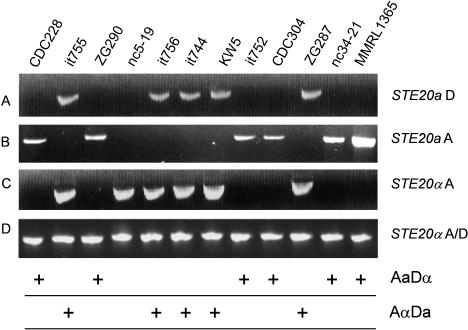
PCR Amplification of Portions of the *STE20* Gene from the AD Hybrid Strains Using Mating Type– and Serotype-Specific Primers (A) PCR primers specific to *MAT**a*** serotype D allele (*STE20D**a***); (B) PCR primers specific to *MAT**a*** serotype A allele (*STE20A**a***); (C) PCR primers specific to *MATα* serotype A allele *(STE20Aα);* (D) PCR primers specific to *MATα* serotype A/D allele *(STE20A/Dα)*. Lanes 1–12 represent the following strains (from left to right): CDC228, it755, ZG290, nc5–19, it756, it744, KW5, it752, CDC304, ZG287, nc34–21, and MMRL1365.

To ascertain the distribution of the mating type alleles in the AD strains, serotype-specific PCR primers were used to amplify fragments of *STE20* gene situated within the *MAT* locus [25]. Five strains, isolated from China, Italy, Kuwait, and the United States of America, generated characteristic products with the PCR primers specific to the serotype A *STE20 MATα* allele (Figure 1C) and serotype D *STE20 MATa* allele (Figure 1A, lanes 2, 5–7, and 10), and therefore were identified as AαD**a** strains (Table 1). Conversely, six strains isolated from China, Italy, and the US produced characteristic amplicons with the PCR primers specific to the serotype A *STE20 MATa* allele (Figure 1B). A pair of PCR primers that were mating-type-specific but non-serotype-specific amplified the *STE20 MATα* gene from all of the strains (Figure 1D). The DNA sequences of these fragments were compared with the available sequences of *STE20* genes for both serotype A [30]. As expected, PCR products obtained from four strains from the US (CDC228, CDC304, MMRL1365, and nc34–21), one strain from China (ZG290), and one strain from Italy (it752) were identical to the reference serotype D *STE20α* sequence, while they exhibited 5% nucleotide polymorphisms with the reference serotype A *STE20α* sequence (Table 2). Thus, they were identified as A**a**Dα strains. An environmental isolate from the US (nc5–19, Figure 1, lane 4) contained two copies of the *STE20α* allele, one from serotype D and the other from serotype A, and therefore had the unusual AαDα genotype, which is consistent with our previous observations ([19] and X. Lin, A. Litvintseva, K. Nielsen, S. Patel, A. Floyd, et al., unpublished data).

**Table 1:**
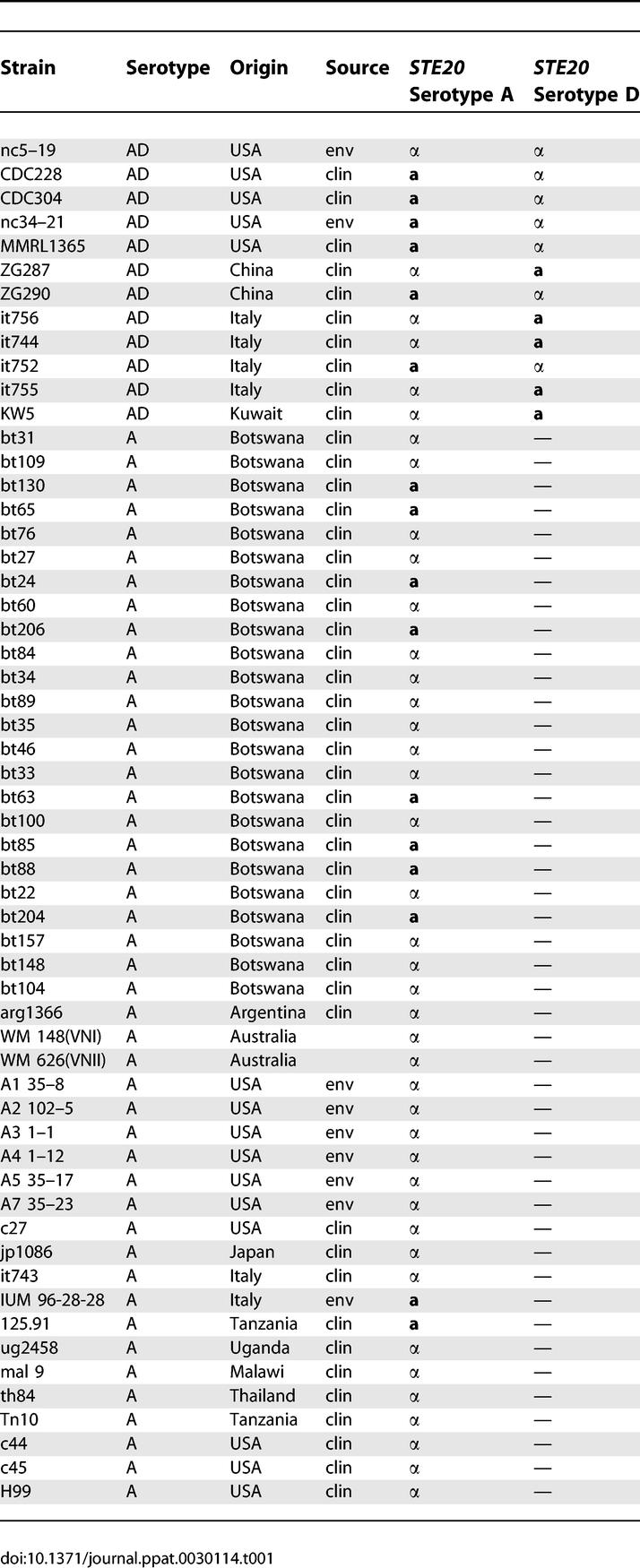
Strains of Cryptococcus neoformans Used in This Study

**Table 2:**
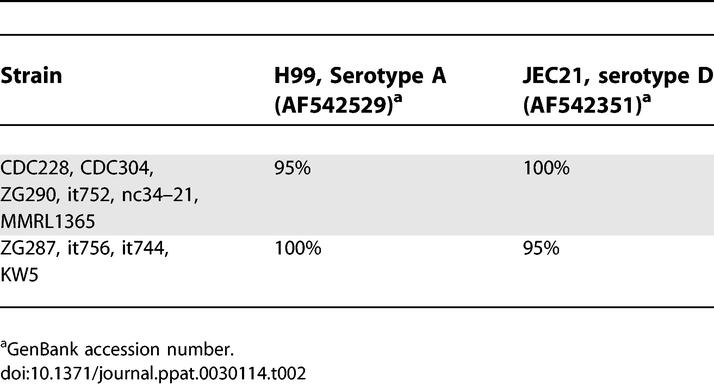
DNA Identity between Portions of *STE20* of Different Strains

### AFLP Genotypes of A**a**Dα and AαD**a** Strains Are Different

**Figure 2:**
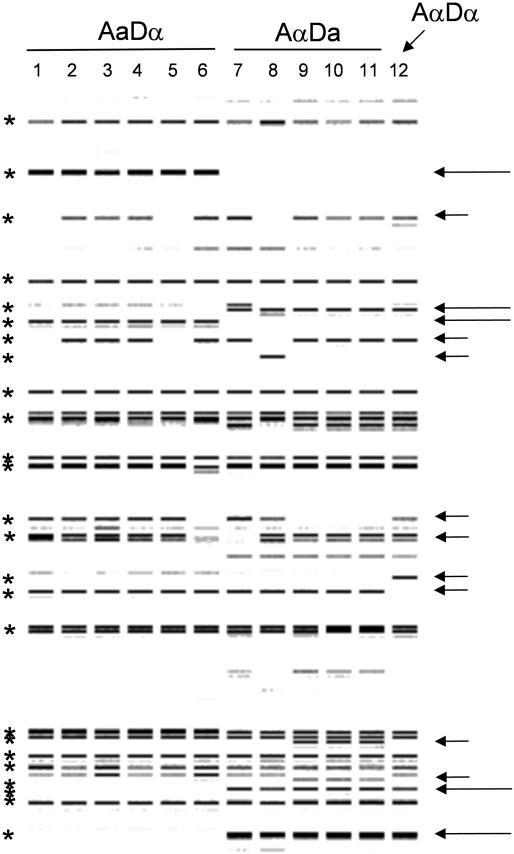
Computer-Generated AFLP Band Patterns of AD Hybrid Strains Lanes 1–6 represent A**a**Dα strains CDC228, CDC304, it752, nc34–21, and MMRL1365, respectively. Lanes 7–12 represent AαD**a** strains ZG287, KW5, it744, it755, it756, and nc5–19, respectively. Lane 12 represents the unusual nc5–19 isolate that carries both serotype A and serotype D *MATα* alleles (AαDα). Bands that are polymorphic between A**a** and Aα groups are indicated with long arrows, and bands that are polymorphic among the individual strains are denoted with short arrows. AFLP bands that were consistent between independent DNA preparations and analyses (see Materials and Methods) are labeled with the asterisks.

To examine the genetic relationships among the 12 AD strains, amplified fragment length polymorphism (AFLP) genotyping was conducted with two independent primer pairs [28]. Eighteen polymorphic bands were generated, which differentiated six unique AFLP genotypes. As shown in Figure 2, AFLP band patterns of the A**a**Dα strains are more similar to each other than to the AαD**a** strains: at least five distinct AFLP bands are characteristic of either one of the groups. Two different genotypes were observed among A**a**Dα strains, and three genotypes were identified among AαD**a** strains. The AFLP banding pattern of the unusual AαDα genotype (strain nc5–19) was similar to that of the AαD**a** strains.

### MLST Analyses Reveal That A**a**Dα Isolates Are Related to VNB Strains from Botswana

**Figure 3:**
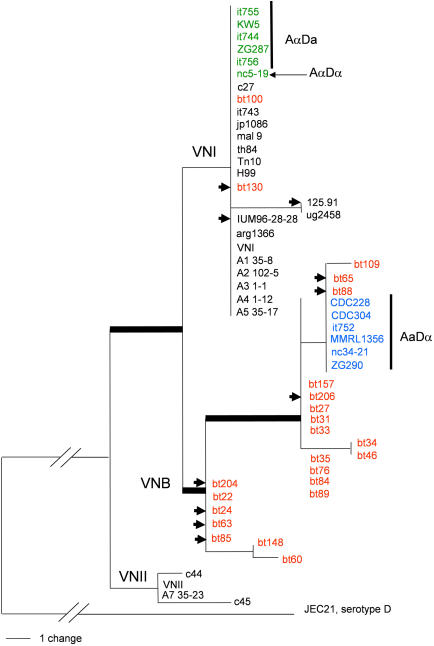
Bayesian Consensus Tree for 12 AD Hybrid Strains and 45 Representative Strains of Serotype A Based on the Combined Data for the Three Loci The tree is rooted with the sequences from the JEC21 strain of serotype D, and branch lengths leading to the outgroup are shortened to fit the figure. Thickened lines denote clades supported by Bayesian posterior probabilities ≥95%. VNI, VNII, and VNB refer to the subgroups (molecular types) identified within serotype A [22]. A**a**Dα hybrid isolates are shown in blue, AαD**a** hybrid isolates are shown in green, and isolates from Botswana are shown in red. Strains of serotype A that possess *MAT**a*** allele are marked with arrowheads.

Previously, we developed a 12-gene multi locus sequence typing (MLST) genotyping system that allows unambiguous genotyping of strains of serotype A; however, this system is impractical for the AD hybrid population, because AD hybrids contain two copies of each loci, both of which are PCR-amplified using our MLST primers [22]. Consequently, we developed new PCR primers that selectively amplified serotype A alleles of three genes, *CAP10, URE1,* and *GPD1,* and generated amplicons of 410, 810, and 220 base pairs, respectively. We used these primer pairs to amplify DNA from the 12 AD hybrids and a global sample of 45 strains of serotype A, which represent unique genotypes in a previously described sample of 1,057 global isolates of serotype A (Table 1, [22]). DNA sequences of these genes were determined and subjected to phylogenetic analyses (Figure 3). For the *CAP10* locus, 308 nucleotides were alignable, and five were phylogenetically informative; for the *URE1* locus, 605 nucleotides could be aligned, and seven were phylogenetically informative; and for the *GPD1* locus, 123 nucleotides were aligned, and three were phylogenetically informative.

Bayesian consensus trees were constructed for *CAP10, GPD1,* and *URE1* (unpublished data), as well as the combined data for all three loci (Figure 3), and rooted with JEC21 strain of serotype D. Gene genealogies of all three loci and the combined genealogy were congruent, and the phylograms recognized the three major subpopulations of serotype A: VNI, VNII, and VNB [22]. The VNI group includes the majority of global isolates of serotype A, as well as three previously identified strains of serotype A with the *MAT**a*** mating type, 125.91 [25], IUM 96-28-28 [26], and bt130 [28]. The VNII group has a small number of strains, and all have the *MATα* allele. The VNB group contains most of the known isolates of serotype A with the *MATa* allele [22]. DNA sequences of the *CAP10, GPD1,* and *URE1* genes were identical among the six A**a**Dα strains. The DNA sequences of these genes were also identical among six AD strains that possess the *MATα* serotype A allele, AαD**a** and AαDα. In all of these phylograms, AαD**a** and AαDα strains cluster with the VNI group, whereas the A**a**Dα strains cluster with the VNB group that contains most of the serotype A *MAT**a*** isolates (Figure 3).

We have not analyzed the origin of the serotype D counterpart in these AD hybrids because an MLST genotyping scheme for serotype D is not yet available. In addition, serotype D isolates are rare in the clinical samples and in the collection, which further complicates this analysis.

### Natural and Laboratory A**a**Dα Hybrids Are More Resistant to UV Irradiation Compared to Serotype A and Serotype D Haploid Strains

Previous analyses of AFLP and MLST genotypes of numerous strains of serotype A showed that the VNB subpopulation is geographically confined to Botswana [22]. Yet, we have shown here that A**a**Dα hybrid strains with the VNB genome are distributed globally. One possible explanation for this apparent paradox is that hybridization produces hybrid strains with increased fitness that are better able to tolerate stress and/or propagate in the environment. Most strains of C. neoformans are highly sensitive to direct sunlight and temperatures above 38 °C, and both conditions exist in much of Botswana. To test the hypothesis of hybrid vigor associated with the AD hybrid genome, we compared the effects of elevated temperature and exposure to UV irradiation on the growth of haploid VNB strains and AD hybrids.

To compare fitness among strains of serotypes A, D, and AD hybrids with the same genetic background, AD hybrids were constructed in the laboratory by fusing *ura5* mutants of bt65 and bt88 strains of serotype A from Botswana, and with an *ade2* mutant of JEC21 strain of serotype D. Spontaneous *ura5* mutants of bt65 and bt88 were obtained by selection on 5-fluoroorotic acid medium, which inhibits strains with the functional *URA5* gene [31,32]. These *ura5* mutants were coincubated with the *ade2* mutant of JEC21 [33] on V8 medium (X. Lin, A. Litvintseva, K. Nielsen, S. Patel, A. Floyd, et al., unpublished data). Three prototrophic hybrid strains, designated XL1595, XL1596, and XL1597, were obtained and confirmed to be diploid by fluorescence-activated cell sorting (FACS) analysis.

**Figure 4:**
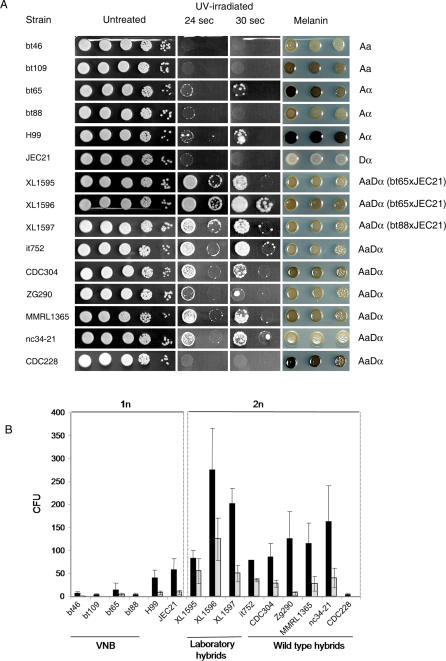
Natural and Laboratory A**a**Dα Hybrids Are More Resistant to UV Irradiation Compared to Serotype A and Serotype D Haploid Strains (A) VNB strains are more sensitive to UV irradiation than isolates of serotype D, VNI strain of serotype A, and AD hybrid strains. Cells were grown overnight in liquid culture under the melanin-inducing conditions, and 10-fold serial dilutions of log-phase yeast cells of VNB strains, AD hybrid strains, and control serotype A and serotype D strains were plated in triplicates on YPD medium; plates were UV irradiated (∼48 mJ/cm^2^) for 24 (second panel) or 30 seconds (third panel). To evaluate the amount of melanin produced, strains were spotted on the plates containing low-glucose L-DOPA medium (last panel). (B) Quantitative assessment of UV resistance for each strain. The highest dilutions that yielded colonies after treatment with UV were identified, and the number of individual colonies was determined within each spot corresponding to the highest dilution that yielded viable colonies. From this number, the total number of CFU that survived the treatment was determined and plotted. The experiment was performed in triplicate, and error bars represent standard error of the mean based on three replicates. Black bars represent cells irradiated for 24 seconds and grey bars represent cells irradiated for 30 seconds. Some AD hybrids are aneuploid (>1n but <2n).

Figure 4 illustrates that VNB isolates of serotype A are more sensitive to UV irradiation than laboratory-generated AD hybrids, naturally occurring AD hybrids, or control haploid strains of serotype A (H99) and serotype D (JEC21). UV irradiation for 24–30 seconds almost completely inhibited the growth of VNB strains and dramatically impaired growth of haploid control strains of serotype A (H99) and serotype D (JEC21); however, a significant number of AD hybrid cells survived this treatment (Figure 4A). The differences in survival between AD hybrids and haploid strains of serotypes A and D were statistically significant (*p* < 0.01, Figure 4B).

Several lines of evidence suggest that C. neoformans is melanized in the natural environment [34,35], and the amount of melanin is known to affect susceptibility of the fungus to environmental stress [36–38]. Therefore, UV irradiation experiments were performed under melanin-inducing conditions. Figure 4A shows that resistance to UV irradiation is not dependent upon the amount of melanin produced by each strain. Although most AD hybrids produced less melanin than haploid strains bt65, bt109, and H99 (Figure 4A, right panel), they were significantly more resistant to UV irradiation (Figure 4A, middle panels). Furthermore, the only heavily melanized hybrid, CDC228, was highly susceptible to UV radiation (Figure 4A, lower right panel).

Recently, we demonstrated that laboratory-generated hybrids with the AαDα genotype are more resistant to elavated temperature than either haploid parent strain (X. Lin, A. Litvintseva, K. Nielsen, S. Patel, A. Floyd, et al., unpublished data). Here, we compared the growth of A**a**Dα hybrids at 40 °C. All of the hybrid strains tested, including laboratory-constructed (XL1595, XL1596, XL1597) and wild-type (ZG290 and nc34–21) hybrid strains, were capable of growing at 40 °C (unpublished data). Two of the haploid strains, JEC21 and bt65, were severely inhibited at 40 °C, whereas strain bt88 was not affected and survived well at 40 °C (unpublished data), which indicates that growth at high temperature can vary among VNB strains. All strains grew equally well on culture medium that contained pigeon excreta [35] as the sole source of carbon and nitrogen (unpublished data).

## Discussion

The high prevalence of the *MAT**a*** serotype A allele among the AD hybrid strains has been puzzling because this allele is uncommon in the global population of serotype A. To investigate the origin of these strains, we designed serotype A–specific PCR primers that amplify three genomic regions situated on three different chromosomes of *C. neoformans (CAP10, GPD1,* and *URE1)* and constructed gene genealogies. These gene genealogies were congruent and revealed that both AαD**a** and AαDα hybrid strains cluster with isolates of the VNI group of serotype A, which is dominated by isolates with the *MATα* mating type. In contrast, A**a**Dα strains cluster with the VNB group from Botswana, which contains a significant proportion of strains with the *MAT**a*** allele [19].

Our results confirm data presented by Xu et al. [39], who used *LAC1* and *URA5* gene sequences to investigate phylogenetic relationships among serotypes A, D, and AD. Their *LAC1* gene phylogeny revealed that AD hybrids are separated into two clusters: one included AD strains possessing the *MAT**a*** serotype A allele, whereas the other included strains of serotype A and AD hybrids possessing the *MATα* serotype A allele. Their *URA5* phylogram confirmed these clusters and also provided evidence of recombination between them, which is consistent with our observations [22,28].

The unusual clinical sample of serotype A isolates from Botswana consists of two genetically isolated subpopulations, VNI-Botswana and VNB [28]. Both groups are genetically unique and characterized by unusually high genotypic diversity. In addition, the VNB group contains an unprecedented proportion of fertile isolates with the *MAT**a*** mating type and exhibits phylogenetic and population genetic evidence of recombination [22,28]. Here, we demonstrate that DNA sequences of the *CAP10, GPD1,* and *URE1* genes in a global collection of A**a**Dα hybrid strains are identical to those of two strains from Botswana, bt65 and bt88 (Figure 3, Table 1), which possess the *MAT**a*** mating type and are fertile in the laboratory [28]. Therefore, it is likely that these, or related Botswanan strains, represent one of the progenitors of the many existing A**a**Dα hybrids.

DNA sequences of the *CAP10, GPD1,* and *URE1* genes were identical among the AD strains with the *MAT**a*** serotype A allele (A**a**Dα), as well as among the six AD strains that possess the *MATα* serotype A allele (AαD**a** and AαDα), which may indicate that the AD hybrids analyzed here are clonal descendents of only three ancestral strains with the A**a**Dα, AαD**a**, and AαDα mating types. However, it is also possible that the loci we used here are insufficiently polymorphic to differentiate among these related strains. For example, we previously analyzed 12 MLST loci in over 100 global isolates of serotype A and demonstrated that two possible parental strains, bt65 and bt88, have closely related, but nevertheless distinct, genotypes. Similarly, this analysis of three MLST loci did not reveal the phylogenetic structure among the VNI isolates that we previously observed using 12 MLST loci and a larger sample of serotype A strains [22]. A**a**Dα and AαD**a** strains are products of hybridization between isolates of opposite mating types *(MAT**a*** and *MATα),* whereas AαDα is an apparent product of mating between isolates of the same mating type, which was recently described in C. neoformans ([40] and X. Lin, A. Litvintseva, K. Nielsen, S. Patel, A. Floyd, et al., unpublished data).

Genetic diversity among the isolates was also estimated by using AFLP, and the phylogenetic analyses based on these data demonstrated that the A**a**Dα strains are more closely related to each other than to strains with the AαD**a** or AαDα genotypes (Figure 2). In addition, AFLP analysis identified two distinct AFLP genotypes among the A**a**Dα isolates, and three distinctive AFLP genotypes among the AαD**a** isolates. The only AαDα isolate (nc5–19) had a unique AFLP genotype that was related to the AαD**a** strains (Figure 2). Both AFLP and MLST analyses suggest at least two, non-exclusive explanations for these data: Strains with identical MLST but distinct AFLP genotypes may have originated from multiple hybridization events between different serotype A and serotype D strains. Conversely, they may have originated from a single hybridization event, but their AFLP genotypes changed following evolution over subsequent generations. For example, some of the AD strains are diploid and others are aneuploid [16]. Therefore, different AFLP genotypes may reflect the loss of chromosomes after the initial hybridization or mitotic recombination and homozygosis. Because both populations of serotype A isolates and AD hybrids exhibit a high level of clonality, contemporary AD strains may be the descendents of a limited number of ancestral AD hybrid strains that proliferated clonally, or may have resulted from multiple hybridization events between clonal strains of serotype A and serotype D. MLST analyses using more loci and careful examination of the karyotypes of these strains are necessary to resolve this question.

Our data indicate that serotype A isolates from the VNB group are geographically restricted to sub-Saharan Africa, whereas A**a**Dα strains that inherited their serotype A genomes from the VNB group are distributed globally. Geographic isolation may have contributed to the unique genetic composition of this Botswanan population. For example, a major portion of Botswana is the Kalahari Desert, which is noted for its remarkably hot, arid climate and high levels of UV radiation. These conditions are likely to deter the spread of VNB strains. This hypothesis is supported by our experiments demonstrating that VNB strains are hypersensitive to UV irradiation and that global A**a**Dα strains of VNB origin are more resistant to UV irradiation and in some cases are more thermotolerant.

Several animal and plant hybrids have been shown to exhibit greater fitness than their parental genotypes in novel or perturbed habitats [1,2]. There are also several cases of the increased fitness of hybrid genotypes among phytopathogenic fungi and oomycetes [41,42]. For example, rapid expansion of interspecies hybrids of the rust fungi, Melampsora medusae and *M. accidentalis,* coincided with the introduction of a new poplar host to the habitat for these fungi, which suggests that these fungal hybrids are better adapted to this new host [7,41]. Similarly, the transmission of lethal infections of alders in Europe has been attributed to the emergence of heteroploid hybrids between Phytophthora cambivora and P. fragariae [4]. Therefore, it is possible that the abundance of the VNB A**a**Dα hybrid strains of C. neoformans in the global population and scarcity of the VNB haploid strains outside Botswana may be attributed to the increased fitness of the hybrids, which enhanced their ability to propagate and disseminate to new environments.

Other explanations for the worldwide distribution of the hybrid strains are also possible. For example, global spread of AD hybrid strains from Botswana may be attributed to human activity and the creation of a novel habitat that reduced competition with the parental species. In particular, an association between C. neoformans and pigeons *(Columbia livia)* is well documented [8,19]. Pigeons were introduced into southern Africa during the period of 1500 to 1700 [43]. The increased availability of pigeon excreta, and perhaps the excreta of other domesticated animals, which are rich substrates for the growth of *C. neoformans,* may have contributed to the global expansion of AD hybrid strains. However, since our experiments demonstrated that all strains grow well on pigeon excreta in the laboratory, other factor(s) may yet be discovered that favor hybrid over haploid VNB strains. It is also possible that VNB isolates will be discovered outside of Africa. For example, if VNB strains were historically more abundant and only recently became restricted to Botswana, hybridization might have occurred outside sub-Saharan Africa. Future analyses of the population structure of the C. neoformans species complex will investigate this possibility.

This investigation confirmed previous observations that approximately half of the global isolates of AD hybrids contain the rare *MAT**a*** serotype A allele ([16,18,39] and X. Lin, A. Litvintseva, K. Nielsen, S. Patel, A. Floyd, et al., unpublished data). We applied methods of AFLP genotyping and phylogenetic analysis of three genes to demonstrate that many A**a**Dα strains possess serotype A genomes from the Botswanan population. We also demonstrated that AD hybrid strains are more resistant to UV irradiation and in some cases are more thermally resistant. These data suggest two hypotheses: (i) A**a**Dα strains probably originated in sub-Saharan Africa from an intervarietal hybridization between isolates of serotype A from the VNB subpopulation and isolates of serotype D; and (ii) this hybridization resulted in increased fitness of the hybrid strain(s), which allowed it to colonize new ecological niches, spread beyond Africa, and populate the world.

## Materials and Methods

### Isolates of C. neoformans.

Twelve AD hybrid strains isolated from four countries were analyzed and compared with a subset of 45 previously described strains of serotype A (Table 1) [22]. Isolates were maintained on yeast extract-peptone-dextrose (YPD) agar medium (Difco, http://www.bd.com/ds/) at 30 °C.

### AFLP.

Genomic DNA was extracted from each isolate and the AFLP analysis was performed as described [28]. Only pronounced and reproducible bands were scored for the analyses of population structure. Polymorphic AFLP bands were defined as bands of the same size that were present in some, but not all, isolates. To assess the reproducibility of the AFLP method, DNA was extracted and the AFLP reactions and analyses were performed on at least two separate occasions for each isolate. In comparing replicate analyses, 92% of the AFLP bands were identical (unpublished data).

### MLST.

We previously developed MLST genotyping for serotype A strains using 12 loci (http://www.cgrubii.mlst.net/). However, for this investigation, we did not amplify and sequence all 12 MLST loci in the AD hybrids, because unlike strains of serotype A or D, which are haploid, the AD hybrids are diploid, and the primers amplified both serotype A and D alleles of the loci. To overcome this problem, we developed new serotype A–specific primers for three of the MLST loci: *CAP10, GPD1,* and *URE1* (PCR primers and conditions are listed in Table 3). Each PCR mixture contained 32 μl of 1X PCR buffer, 2 mM MgCl_2_, 0.2 mM dNTPs, 1 μM each primer, 0.065 μl of i*Taq* DNA Polymerase (Bio-Rad, http://www.bio-rad.com/), and approximately 1 ng of genomic DNA. PCR products were purified using the QIAquick PCR purification kit (Qiagen, http://www.qiagen.com/) and sequenced using an ABI 3700 sequencer with Big Dye terminators (Applied Biosystems, http://www.appliedbiosystems.com/). PCR primers used for the amplification of the fragments were also used for sequencing.

**Table 3:**
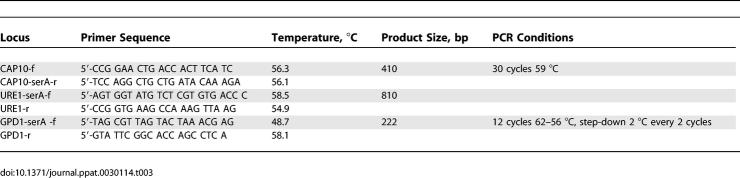
PCR Primers

### Mating type identification.

Mating type– and serotype-specific primers were used to confirm the mating type alleles of the AD hybrid strains. The primer pairs, which amplify portions of the *STE20**a*** or *STE20α* alleles of serotype A or D, are designated *STE20A**a**, STE20Aα,* and *STE20D**a**,* respectively [25]. Since the primers that were previously designed to amplify a serotype D–specific portion of the *STE20α* gene failed to generate any product in the AD strains, we designed new PCR primers specific to a more conserved region of the *STE20α* gene of serotype D: *STE20α-F*: 5′-GTAAGTGCAAAGGACCCATCTC; and *STE20α-R*: 5′-TGATCC CCAAAGACCAAATATC. The PCR conditions for amplification were as follows: 94 °C for 5 min, followed by 30 cycles of 94 °C for 30 sec, 52 °C for 30 sec, and 72 °C for 1 min, followed by 7-min extension at 72 °C (PCR reactions were the same as those described for MLST). DNA sequences of the corresponding *STE20α* portions were obtained and compared with the DNA sequences in the typing strains, JEC21, serotype D [30], and H99, serotype A (C. neoformans H99 sequencing project, Duke Institute for Genome Sciences and Policy, Center for Applied Genomics and Technology, http://cgt.duke.edu/).

### Data analyses.

Sequences were automatically aligned using Sequncher 4.1 (Gene Codes Corporation, http://www.genecodes.com/); the alignment was imported into MacClade 4.05 [44] and manually edited. Ambiguously aligned characters and gaps were excluded from the analysis. Optimal phylogenetic trees were constructed using maximum parsimony (MP) and Bayesian approaches. MP trees for the individual loci and for the combined data set were identified with heuristic searches based on 500 random sequence additions for each data set and implemented in PAUP version 4.0b10 [45].

Bayesian inferences were performed with MrBayes version 3.0B4 using a General Time Reversible model with a proportion of invariable sites and a gamma-shaped distribution of rates across sites (GTR+I+G) model of evolution [46]. Each Bayesian analysis consisted of two runs of 1,000,000 generations, each using the default uniform priors, and a sample frequency of 100. Likelihood scores of each sampled generation were plotted by using Excel (Microsoft, http://www.microsoft.com/) and visually analyzed; phylograms collected before the stationary phase was reached were discarded [47]. The phylograms remaining from both runs were combined, and a majority-rule consensus phylogram of each gene and for the combined data from all three genes were generated using PAUP and compared for topological congruence.

### Construction of A**a**Dα hybrid strains.

To construct A**a**Dα hybrid strains in the bt65/bt88 and JEC21 background, *ura5* mutants of strains bt65 and bt88 were obtained. Wild-type strains bt65 and bt88 were grown in liquid YPD, washed three times in sterile distilled water, and plated on 5-fluoroorotic acid (5-FOA) medium, which inhibits cells with a functional *URA5* gene [32]. 5-FOA-resistant colonies were selected and tested on the yeast nitrogen base minimal medium (YNB, Difco). Isolates that showed no growth on YNB (A**a** *ura5*) were mixed with cells of the XL342 (Dα *ade2*) strain of serotype D [33] and incubated on V8 agar medium in the dark at 22 °C. After coincubation for 24 h, cells were collected, washed, and spread on YNB minimal medium to select for prototrophic fusion products. The ploidy of prototrophic A**a**Dα strains was confirmed by FACS analysis.

### Ploidy determination by FACS*.*

Yeast cells were processed for flow cytometry as described previously [48]. Briefly, cells were harvested from YPD medium, washed once in PBS buffer, and fixed in 1 ml of 70% ethanol overnight at 4 °C. Fixed cells were washed once with 1 ml of NS buffer (10 mM Tris-HCl [pH 7.6]; 250 mM sucrose; 1 mM EDTA [pH 8.0]; 1 mM MgCl_2_; 0.1 mM CaCl_2_; and 0.1 mM ZnCl_2_) and then stained with propidium iodide (10 mg/ml) in 0.2 ml of NS buffer containing RNaseA (1 mg/ml) at 4 °C for 4–16 h. Then 0.05 ml of the stained cell preparation was diluted into 2 ml of 50 mM Tris-HCl (pH 8.0) and sonicated for 1 min. Flow cytometry was performed on 10,000 cells and analyzed on the FL1 channel of a Becton-Dickinson FACScan (http://www.bd.com/).

### Sensitivity to UV irradiation.

The following strains were tested for their sensitivity to UV irradiation: (i) six wild-type A**a**Dα hybrid strains; (ii) three A**a**Dα hybrids obtained in the laboratory by fusing bt65/bt88 and JEC21 strains; (iii) four strains of serotype A from the VNB subpopulation, including the two strains (bt88 and bt65) that were most closely related to the probable serotype A progenitor of A**a**Dα strains; and (iv) the representative haploid strain of serotype A (H99) and representative haploid strain of serotype D (JEC21). All strains were grown to logarithmic phase at 30 °C with constant agitation in the dark in low-glucose medium (0.1% glucose, 1.3 mM glycine, 2.2 mM KH_2_PO_4_, 0.1 mM MgSO_4_-7H_2_O, 0.3 μM thiamine, 2 nM biotin, [pH 5.6]) supplemented with DL-3,4-dihydroxyphenylalanine-(DL-DOPA, 20mg/l). Yeast cells were counted in a hemocytometer, all cultures were adjusted to the same cell concentration (5.5 × 10^7^ CFU/ml), serial 10-fold dilutions were prepared, and 2.0 μl of each strain was spotted onto YPD agar plates (to assess sensitivity to the UV irradiation) and low-glucose agar plates supplemented with 200 mg/l DL-DOPA (to assess melanin production). The YPD plates were then irradiated with UV light at approximately 48 mJ/cm^2^ (24- and 30-second settings, UV Stratalinker 1800; Stratagene, http://www.stratagene.com/) [49], and all plates were incubated at 30 °C for 72 h. To provide a quantitative assessment of UV resistance for each strain, the highest dilutions that yielded colonies after treatment with UV were identified. For such dilution, the number of individual colonies within a spot on the YPD plate was counted.

## Supporting Information

### Accession Numbers

The GenBank (http://www.ncbi.nlm.nih.gov/Genbank/index.html) accession numbers for the *STE20* genes discussed in this paper are H99 serotype A (AF542529) and JEC21 serotype D (AF542531).

MLST genotyping for serotype A strains using 12 loci is under accession numbers DQ212527–DQ212692. Unique MLST sequence types were deposited under accession numbers EF625826–EF625831.

## Abbreviations

AFLP: amplified fragment length polymorphism
FACS: fluorescence-activated cell sorting
MLST: multi locus sequence typing
